# Evaluation of Electroencephalography Source Localization Algorithms with Multiple Cortical Sources

**DOI:** 10.1371/journal.pone.0147266

**Published:** 2016-01-25

**Authors:** Allison Bradley, Jun Yao, Jules Dewald, Claus-Peter Richter

**Affiliations:** 1 Department of Biomedical Engineering, Northwestern University, Evanston, IL, United States of America; 2 Department of Otolaryngology, Feinberg School of Medicine, Northwestern University, Chicago, IL, United States of America; 3 Department of Physical Therapy and Human Movement Science, Feinberg School of Medicine, Northwestern University, Chicago, IL, United States of America; 4 Department of Physical Medicine and Rehabilitation, Northwestern University, Chicago, IL, United States of America; 5 Hugh Knowles Center, Department of Communication Sciences and Disorders, Northwestern University, Evanston, IL, United States of America; University of Rome, ITALY

## Abstract

**Background:**

Source localization algorithms often show multiple active cortical areas as the source of electroencephalography (EEG). Yet, there is little data quantifying the accuracy of these results. In this paper, the performance of current source density source localization algorithms for the detection of multiple cortical sources of EEG data has been characterized.

**Methods:**

EEG data were generated by simulating multiple cortical sources (2–4) with the same strength or two sources with relative strength ratios of 1:1 to 4:1, and adding noise. These data were used to reconstruct the cortical sources using current source density (CSD) algorithms: sLORETA, MNLS, and LORETA using a p-norm with p equal to 1, 1.5 and 2. Precision (percentage of the reconstructed activity corresponding to simulated activity) and Recall (percentage of the simulated sources reconstructed) of each of the CSD algorithms were calculated.

**Results:**

While sLORETA has the best performance when only one source is present, when two or more sources are present LORETA with p equal to 1.5 performs better. When the relative strength of one of the sources is decreased, all algorithms have more difficulty reconstructing that source. However, LORETA 1.5 continues to outperform other algorithms. If only the strongest source is of interest sLORETA is recommended, while LORETA with p equal to 1.5 is recommended if two or more of the cortical sources are of interest. These results provide guidance for choosing a CSD algorithm to locate multiple cortical sources of EEG and for interpreting the results of these algorithms.

## Introduction

Source localization algorithms are commonly used to find the cortical sources of scalp recorded EEG (for introductory reviews of EEG source localization see [1–3]). Multiple areas of the cortex are often expected to be active at the same time. Current source density (CSD) models were developed to overcome the limitations of dipole models, which assume a single or a small number of dipoles can represent the source of EEG [4]. CSD reconstructions often show activity in multiple areas of the cortex. For example, a literature search of the Medline database returned 15 journal articles that reported multiple active areas of the cortex found with source localization algorithms published in November 2013 alone. Yet, the use of commonly used source localization algorithms for the detection of multiple active areas has not been validated. Most studies evaluating source localization algorithms do so by using single source areas, not multiple distinct areas (e.g. [5–8]). Assuming that results showing multiple active areas are as accurate as single source areas is not necessarily valid. Algorithms may miss some sources entirely, or may locate activity where there is none. This lack of data on the performance of source localization algorithms in locating multiple sources makes choosing an algorithm, and interpreting the results of an algorithm, difficult. This issue is particularly important as there are often multiple sources of EEG, and distinguishing these sources can give important insight into the workings of the brain. For example, when recording motor related potentials, various cortical regions may be active simultaneously such as during visually guided movements [9] or while performing motor tasks requiring mental image transformations [10]. Accurately reconstructing this activity would allow for the study of how cortical regions are associated with various motor tasks.

The aim of this study is to empirically compare source localization algorithms when locating multiple simultaneously active cortical generators, with equal or different strengths. Specifically, this paper focuses on common algorithms that use the current source density (CSD) model, rather than a dipole model. The methods tested in this study include low resolution electromagnetic tomography (LORETA) [11, 12] using a p-norm with p equal to 1, 1.5, and 2, minimum norm least square (MNLS) [13], and standardized low resolution electromagnetic tomography (sLORETA) [14]. Although LORETA with a p-norm <2 is not commonly used, these variants were included in this study as they showed superior performance in a previous study [15].

In a previous study, the localization accuracy and resolution of LORETA, MNLS and dipole methods were compared when localizing one cortical source and two sources with varying size and separation [15]. In that study, LORETA using a p-norm with p equal to 1 was found to have the best localization accuracy and highest resolution. Grova et al. (2006) also compared the performance of current source density source localization algorithms with one and two sources, and found LORETA and maximum entropy on the mean approaches best reconstructed the simulated sources [6]. Wagner et al. (2004) compared the performance of the sLORETA algorithm to that of LORETA and MNLS using two simulated dipole sources with varying locations and orientations [16]. For the eleven locations and three orientations tested, they found that none of the methods separated the sources when the dipoles had similar orientations, and could not detect a weak source deep in the brain in the presence of a stronger, more superficial one. However, as many of the sources in that study were deep within the brain, these results may not reflect the accuracy of locating cortical sources, which, originating close to the scalp, are often easier to record and locate. Similarly, Grave de Peralta Menendez and Gonzalez Andino (2001) evaluated MNLS, LORETA and another averaged solution algorithm called LAURA and found that all performed similarly with sources distributed throughout the brain, but performed better for cortical sources than for deeper sources [17].

**Fig 1:**
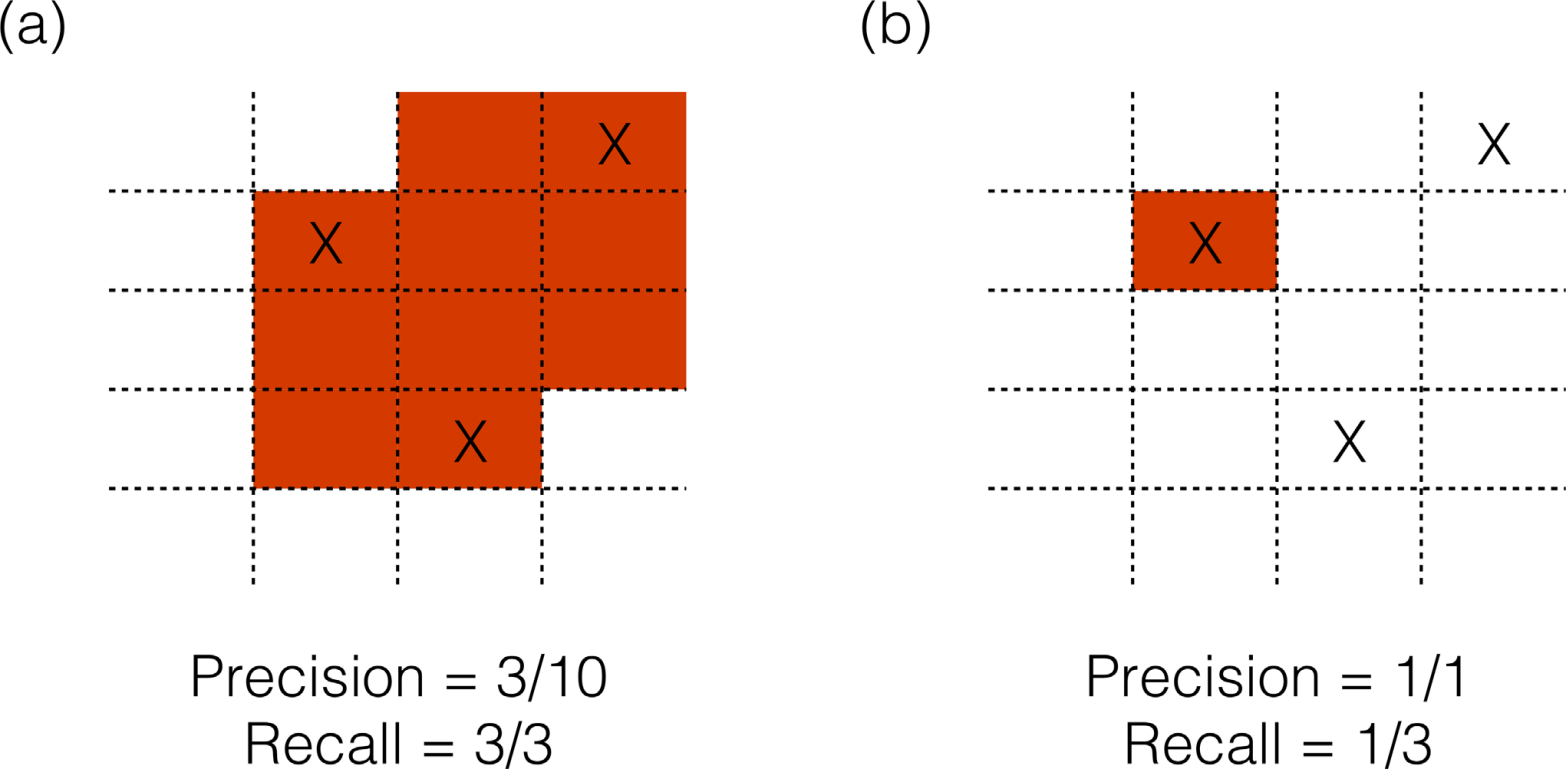
Schematic example of Precision and Recall calculation. Real source locations are represented by “X” marks. Red shading represents a hypothetical source reconstruction. (a) If an algorithm designates a large area as active, it is likely to find all sources, but the Precision of the reconstruction will be low. (b) If an algorithm is more conservative, Precision is high, but Recall is low as not all sources are found.

As in previous studies, the source localization algorithms were tested with simulated sources. One to four simultaneously active sources were simulated only on the cortex, with orientation perpendicular to the surface of the cortex. The locations of the sources were varied to minimize the dependence of result on one source location, and the effect of varying the strength of one of the sources was also evaluated. To compare different algorithms, two measures of localization performance were adapted from the information retrieval and machine learning literature [18]–Precision, which measures how many of the reconstructed sources were real sources, and Recall, which measures what fraction of the real sources were found by the inverse algorithm. As shown in Fig 1A, if an algorithm designates a large area as active, it is likely to find all sources, but the Precision of the reconstruction will be low; and in Fig 1B if an algorithm is more conservative, the Precision is high, but Recall is low as not all sources are found. Together, these two measurements summarize the ability to accurately identify cortical generators of scalp-recorded activity, while minimizing false positives i.e. falsely locating activity in areas that are not actually active.

The results of this study provide data on the accuracy to expect from the CSD source localization methods tested, and guidance on the source localization algorithm to choose for the purpose of detecting multiple sources.

## Methods

### 2.1 Simulation of EEG scalp recordings

#### 2.1.1 Head model

A 3-shell boundary element method (BEM) head model [19] based on a real, high-resolution head MRI was used to represent the geometry of the brain, skull and scalp. This MRI was obtained for a separate study, for which the participant gave written, informed consent. Both the original recording of the MRI and the use of the MRI data for this study were approved by the Institutional Review Board of Northwestern University. The MRI data were anonymized immediately after recording.

The head model was created using the Curry V5.0 (Compumedics Neuroscan, Charlotte, NC) software package. The resolution of the layers of the BEM model were: skin 10 mm, skull 9 mm, brain 7 mm. The conductivities assigned to the layers were: skin 0.33 S/m skull 0.0174 S/m and brain 0.33 S/m [20]. A model of the cortex served as the source space, with a resolution of 3mm. The same head model was used for the calculation of scalp potentials from the simulated sources (the forward calculation) and the reconstruction of activity at the cortex from these scalp potentials (the inverse calculation). Using the same head model for the forward and inverse calculation minimized errors in localization due to inaccuracies in the head model.

#### 2.1.2 Positions of EEG electrodes

The locations of 160 EEG electrodes used in the simulations were taken from a real EEG experiment with the same subject as the MRI images. The position of each electrode was recorded using a 3D magnetic digitizer system (Polhemus, Colchester, VT). Furthermore, anatomical landmarks, including the nasion and 2 preauricular points, were digitized. Using these anatomical landmarks, the electrode locations were co-registered with the MR images and included in the head model.

#### 2.1.3 Simulation of cortical sources

The model of the cortex surface (constructed in the Curry software with the head model, see section 2.1.1) was used as the source space for the simulations of the cortical sources. A location on the model of the cortex was chosen at random, and a dipole was placed at that point and each of the four points closest to it on the model of the cortex. As the resolution of the cortex surface was 3mm each source had an approximate surface area of 25 mm^2^. Each dipole was oriented perpendicular to the cortex surface, in alignment with the pyramidal cells thought to generate cortical potentials [21].

#### 2.1.4 Simulation of surface EEG measured at scalp

Using the BEM head model described above, the scalp potentials corresponding to each of the cortical sources were first calculated individually. To simulate multiple sources, 2–4 of these simulated scalp potentials were averaged together. Similarly, to simulate sources of different strengths, two simulated scalp recordings were averaged with different weights to give strength ratios of 1:1 to 1:4. To reduce the impact of a specific source location on the inverse algorithm evaluation, for each condition 250 different surface EEG signals were simulated using 250 combinations of sources located at different places on the cortex. For sources of different strengths, the sources were kept in the same location for each strength ratio and only their strengths were varied. Finally, white noise was added to the simulated scalp recordings to give a maximum signal to noise ratio (SNR) of 10. Maximum SNR was calculated as the maximum simulated scalp potential amplitude divided by the amplitude of the added noise.

#### 2.2 Current Source Density Reconstruction

Each of the CSD algorithms, including MNLS, sLORETA and LORETA using a p-norm with p equal to 1, 1.5 and 2 (subsequent references to the LORETA algorithm will be referred to as LORETA 1, LORETA 1.5 and LORETA 2), were used to reconstruct the cortical current distribution from the simulated scalp recordings. For the LORETA algorithm, the p-norm was altered on both the model and data terms. The algorithms tested are commonly used CSD algorithms that are available in the commercially available Curry V5 software package (Compumedics Neuroscan, Charlotte, NC). No location weighting was used in any of the reconstruction. For each algorithm, the regularization parameter (lambda), which defines the trade-off between matching the scalp recordings and matching the constraints of the particular algorithm, was adjusted automatically by the Curry software. The Curry software searches for a lambda that results in a residual deviation approximately equal to the variance in the data due to noise – i.e. 1/SNR. In our case, with an SNR of 10, we were searching for a lambda term that would give 9.5–10.5% residual deviation.

### 2.3 Evaluation of CSD results

The output of the CSD algorithms is a list of voxels on the cortex (set during the head model creation, and in our case 3mm apart) that have been assigned a current strength value (or in the case of sLORETA an f-statistic that represents the probability of that voxel being active). Source localization algorithms have two competing goals: reconstructing all sources and minimizing false reconstruction in areas where there is no source. An algorithm can detect all the sources present if it labels every voxel as a source (Fig 1A). This would result in localization of all the actual sources but generate a very high false detection rate. On the other extreme, an algorithm could be tuned to be conservative and only label one voxel as a source, correctly (Fig 1B). That would make the algorithm precise but would also miss detecting most of the sources present. Therefore, as discussed in section 1 above, we adapt two metrics used for this purpose–Precision and Recall–from the field of information retrieval and pattern recognition and use them as evaluation measures for this study [18]. Precision and Recall analysis is similar to the ROC analysis used by Grova et al. [6] but is more suited to situations where the solution space is expected to be sparse, as expected with the sources of EEG.

For calculation of Precision and Recall ‘active’ voxels were defined as voxels whose strength was higher than a strength threshold. Strength thresholds from 5% to 95% of the maximum source strength of the reconstruction were used. The threshold was varied to evaluate the impact of the threshold on the performance of the algorithms. As source reconstructions are known to have some “blurring” around the source locations, active voxels with a distance from the closest real source of less than 10 mm were counted as “correct”. Precision was calculated as the number of “correct” voxels divided by the total number of ‘active’ voxels. Recall was calculated as the number of real sources that had an “active” voxel within 10 mm, divided by the total number of real sources (1–4) in the simulation. Precision and Recall were calculated for each strength threshold for each of the 250 source locations in each condition and the average Precision and Recall calculated for each condition. As Precision and Recall are complimentary metrics, in addition to looking at the Precision and Recall plotted against strength threshold, we plot Precision against Recall (known as PR curves) and calculate the area under that curve (known as AUC) as an additional performance metric that balances the two metrics and gives an overall picture of the performance of the algorithm. The AUC for each of the 250 source locations in each condition was calculated individually.

The statistical significance of differences in AUC between algorithms for each condition (number of sources, strength of sources) was assessed with Friedman’s non-parametric test, implemented in MATLAB (The MathWorks, Inc., Natick, Massachusetts, United States). Each of the 250 AUCs were included for each condition. Post-hoc analysis with a Tukey-Kramer correction determined if the difference between the highest performing algorithm and its closest competitor was significant.

## Results

**Fig 2:**
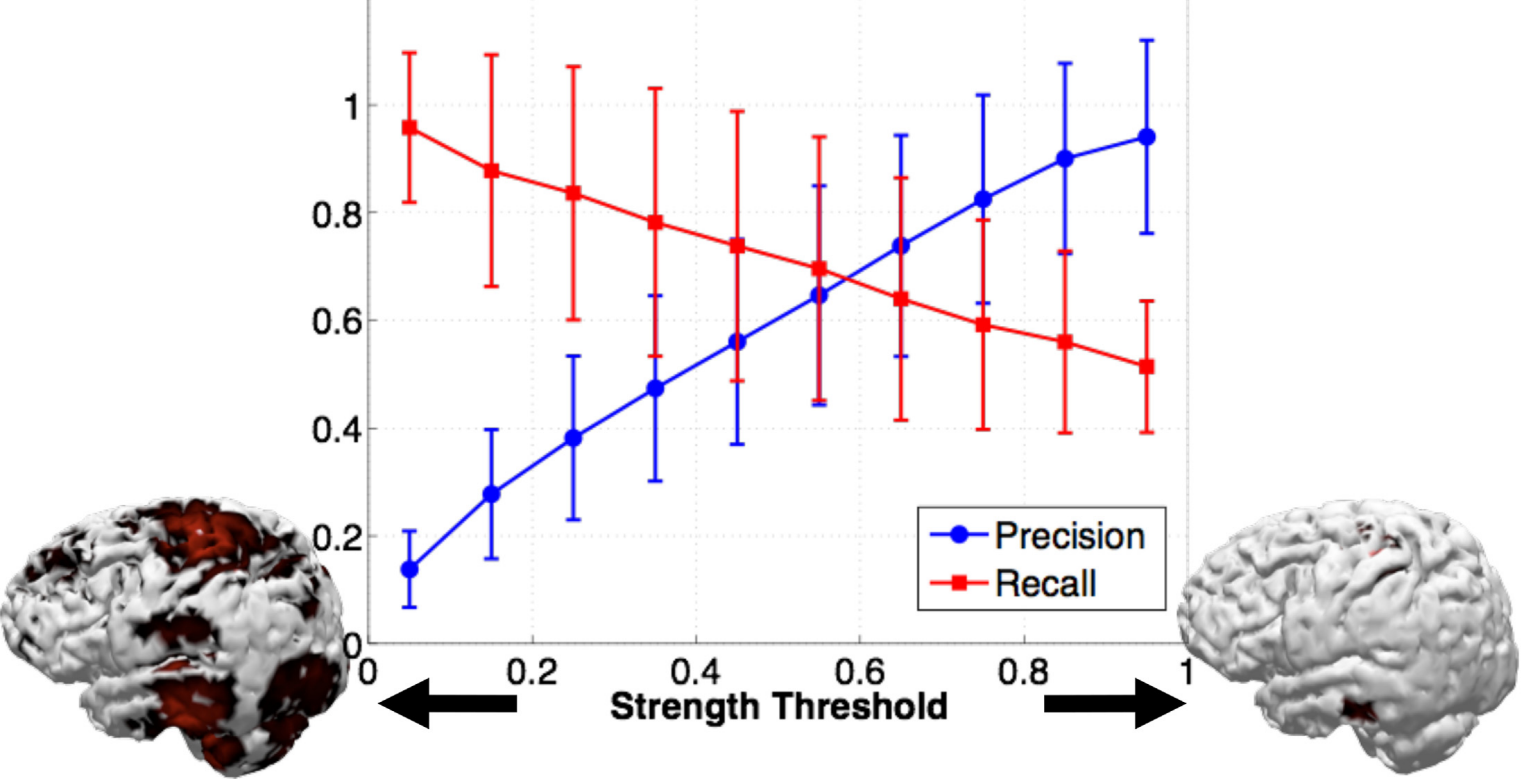
An example of Precision (blue) and Recall (red) plotted against strength threshold, for the sLORETA algorithm and 2 simulated sources. The picture of the cortex on the left indicates what imposing a low (5%) strength threshold on the CSD result looks like, with widespread activity. The cortex on the right indicates what imposing a high strength threshold (95%) looks like, with sparse activity.

An example of the Precision and Recall results obtained over the range of strength thresholds is shown in Fig 2. In this example, two simulated sources were reconstructed with sLORETA, but the relationship between strength threshold and Precision and Recall is similar for all algorithms. As expected, with increasing strength threshold, the algorithms become more conservative and only label a smaller area as active. This results in an increase in Precision with increasing strength threshold, but a decrease in Recall, as fewer of the sources are likely to be found. All reconstructions follow this general trend, with only the peak Precision and Recall values and the slopes changing. Therefore, in order to make the algorithm comparison clearer, the remainder of our Precision and Recall results are presented as plots of Precision versus Recall, omitting the strength threshold relationship. Note that an ideal algorithm would have a perfect Precision of 1 while simultaneously having a perfect Recall of 1, giving an area under the Precision-Recall curve equal to 1.

**Fig 3:**
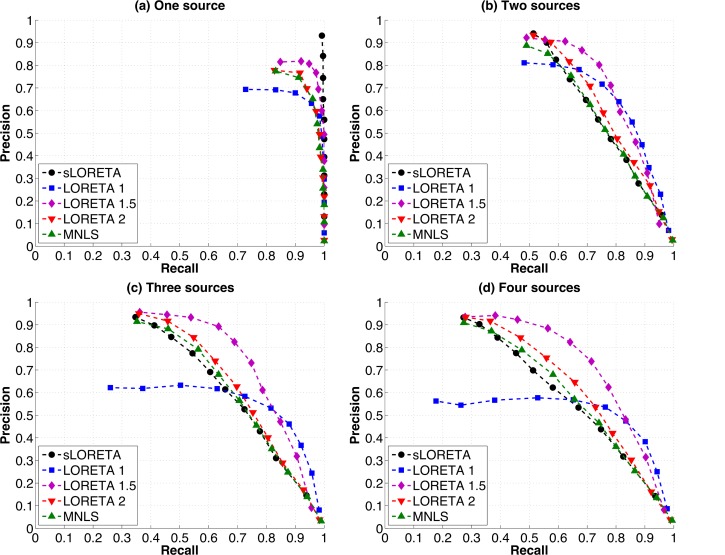
Precision vs. Recall of each of the source localization algorithms (MNLS, sLORETA, LORETA 1, 1.5 and 2) for (a) one, (b) two, (c) three and (d) four simulated sources. Each point indicates the Precision and Recall for a different strength threshold. For one source (a) sLORETA simultaneously has Precision and Recall values of close to 1, indicating perfect performance, while other algorithms have lower peak Precision. For two sources (b) all algorithms reach peak Precision only when Recall has been reduced to about 0.5, and peak Recall is reached only when Precision has dropped below 0.3. For three sources (c) LORETA 1.5 has higher Recall for many Precision values than other algorithms, LORETA 1 has lower peak Precision, while all other algorithms follow a similar pattern, with high Precision that drops off steeply with increasing Recall. For four sources (d) while peak Precision is still high for most algorithms, Recall drops slightly.

When there is only one source active (Fig 3A), all algorithms perform well, finding the source with little falsely detected activity. sLORETA performs the best, with an almost perfect Precision of 0.93 and Recall of 1 corresponding to high strength thresholds. Other algorithms have lower peak Precision: LORETA 1 has a peak Precision of 0.69, LORETA 1.5 has a peak Precision of 0.81, LORETA 2 has a peak Precision of 0.78 and MNLS has a peak Precision of 0.77. Perfect Recall is possible with all algorithms, but only sLORETA has perfect Recall for all points on the graph, corresponding to all strength thresholds.

When two sources are active simultaneously (Fig 3B), the trade-off between Precision and Recall becomes clear, as for all algorithms Precision is high (>0.8) when Recall is lowest (0.45–0.55), and vice versa. A Recall of 0.5 means that only one of the two sources is being accurately located. Note that peak Precision is higher for two sources than for one source (0.94 for sLORETA, 0.81 for LORETA 1, 0.92 for LORETA 1.5, 0.93 for LORETA 2 and 0.89 for MNLS). This is likely due to an increased area being counted as correct for two sources than for one. However, peak Precision decreases quickly as Recall increases. For example, sLORETA has a higher peak Precision (0.94) than other algorithms when one source is found, but this Precision decreases as the likelihood of finding the second source increases. When recall has reached 0.75 (corresponding to a 50% chance of finding the second source), sLORETA has amongst the lowest Precisions (~0.5).

When the number of sources is increased to three (Fig 3C), MNLS, sLORETA and LORETA 1.5 and 2 converge on a high peak Precision (0.93 for sLORETA, 0.91 for MNLS, 0.96 for LORETA 1.5, 0.95 for LORETA 2) while LORETA 1 has a lower Precision for most strength thresholds, with a peak of 0.62. Again, high Precision is only possible when Recall is at a level where only one source is found, and Precision drops off quickly as the probability of finding the second and third source increases. sLORETA Precision drops off slightly more steeply than for other algorithms.

With four sources (Fig 3D), LORETA 1 once again has lower peak Precision (0.56) than other algorithms (0.93 for sLORETA, 0.91 for MNLS, 0.93 for LORETA 1.5, 0.93 for LORETA 2). LORETA 1.5 and LORETA 1 have higher Recall at many points than other algorithms, as their Precision does not decay as quickly with increasing Recall. Again, sLORETA Precision decays more steeply as Recall increases.

**Fig 4:**
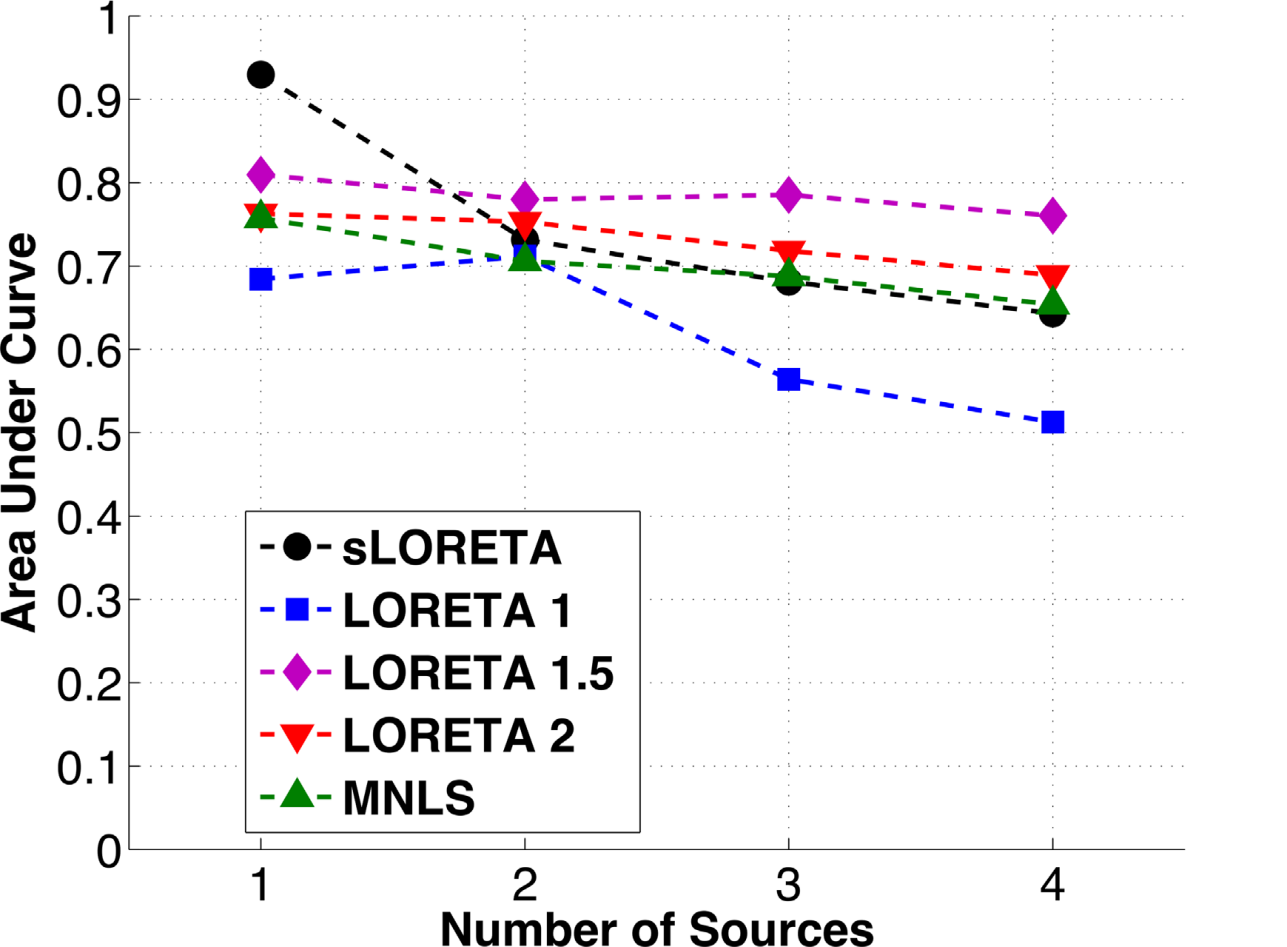
Area under the Precision vs. Recall (PR) curve for each of the algorithms tested vs. the number of sources in the simulation. The area under the PR curve serves as a summary of the overall performance of the algorithm. Note that while sLORETA is clearly the best algorithm when there is only one source present, its performance drops off as the number of sources increases. The performance of all the other algorithms appears steady for 1–4 sources, as Precision increases slightly for these algorithms while Recall drops.

Overall, the area under the Precision-Recall curve remains approximately the same across numbers of sources tested for all of the algorithms except for sLORETA, which has a sharp decrease in performance between one and two sources, and LORETA 1, which has a steady decrease in performance between two, three, and four sources (Fig 4). The steady AUC performance for LORETA 1.5, LORETA 2 and MNLS is due to a decrease in Recall being balanced by an increase in Precision (as the area of the cortex counted as “correct” is increased). While sLORETA clearly has superior performance for one source, it does not do as well when the number of sources increases. For three and four sources LORETA 1.5 has higher area under the curve than all other algorithms, owing to its higher Recall values. The results of a Friedman test of the statistical significance of differences in AUC between algorithms are presented in Table 1, in addition to post-hoc testing of the difference between the top two performing algorithms. The differences between the top performing algorithm and its closest competitor were significant at the level p = 0.1 for all conditions.

**Table 1:** Results of Friedman test of statistical significance of differences in AUC.

Friedman test				Post-hoc test		
	Highest AUC algorithm	Second highest AUC algorithm	Significance of difference	Highest AUC Algorithm	Second highest AUC algorithm	Significance of difference
One source	sLORETA	LORETA 1.5	p<0.01	sLORETA	LORETA 1.5	p<0.01
Two sources/ 1:1 strength ratio	LORETA 1.5	LORETA 1	p<0.1	LORETA 1.5	LORETA 1	p<0.1
Three sources	LORETA 1.5	LORETA 2	p<0.001	LORETA 1.5	LORETA 2	p<0.001
Four sources	LORETA 1.5	LORETA 2	p<0.001	LORETA 1.5	LORETA 2	p<0.001
2:1 strength ratio	LORETA 1.5	LORETA 2	p<0.001	LORETA 1.5	LORETA 2	p<0.001
3:1 strength ratio	LORETA 1.5	LORETA 2	p<0.001	LORETA 1.5	LORETA 2	p<0.001
4:1 strength ratio	LORETA 1.5	LORETA 2	p<0.001	LORETA 1.5	LORETA 2	p<0.001

**Fig 5:**
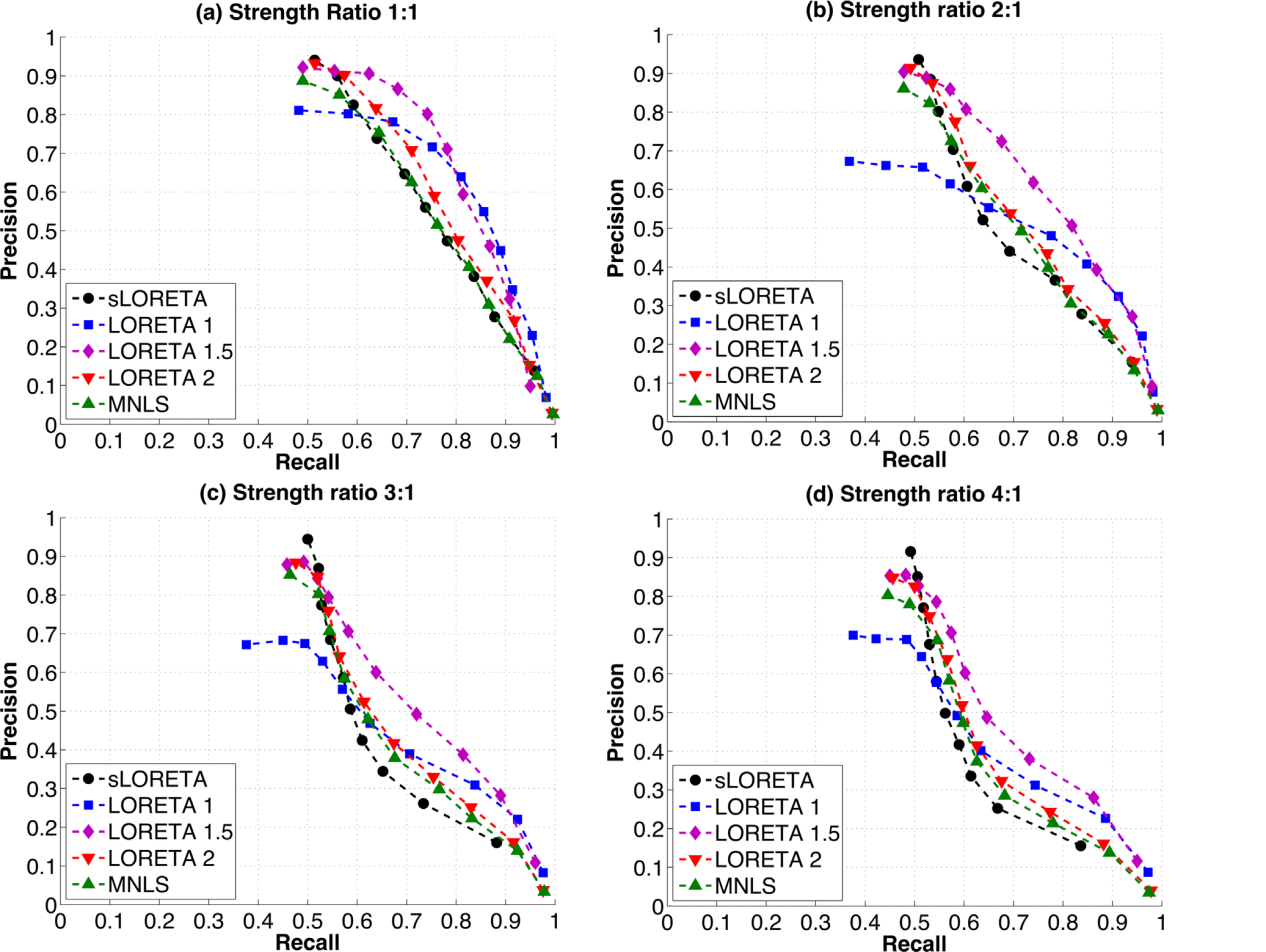
Precision vs. Recall curves for two sources with varying strength ratios: (a) two sources with the same strength (b) two sources, one with twice the strength as the other (c) two sources, one with three times the strength as the other (d) two sources, one with four times the strength as the other. Note that while sLORETA has highest peak Precision for all strength ratios, Precision also drops off more steeply with increasing Recall for this algorithm. This pattern is emphasized as the strength ratio increases. All other algorithms have similar performance to each other, with slightly decreasing Precision and Recall as the strength ratio of the sources increases.

**Fig 6:**
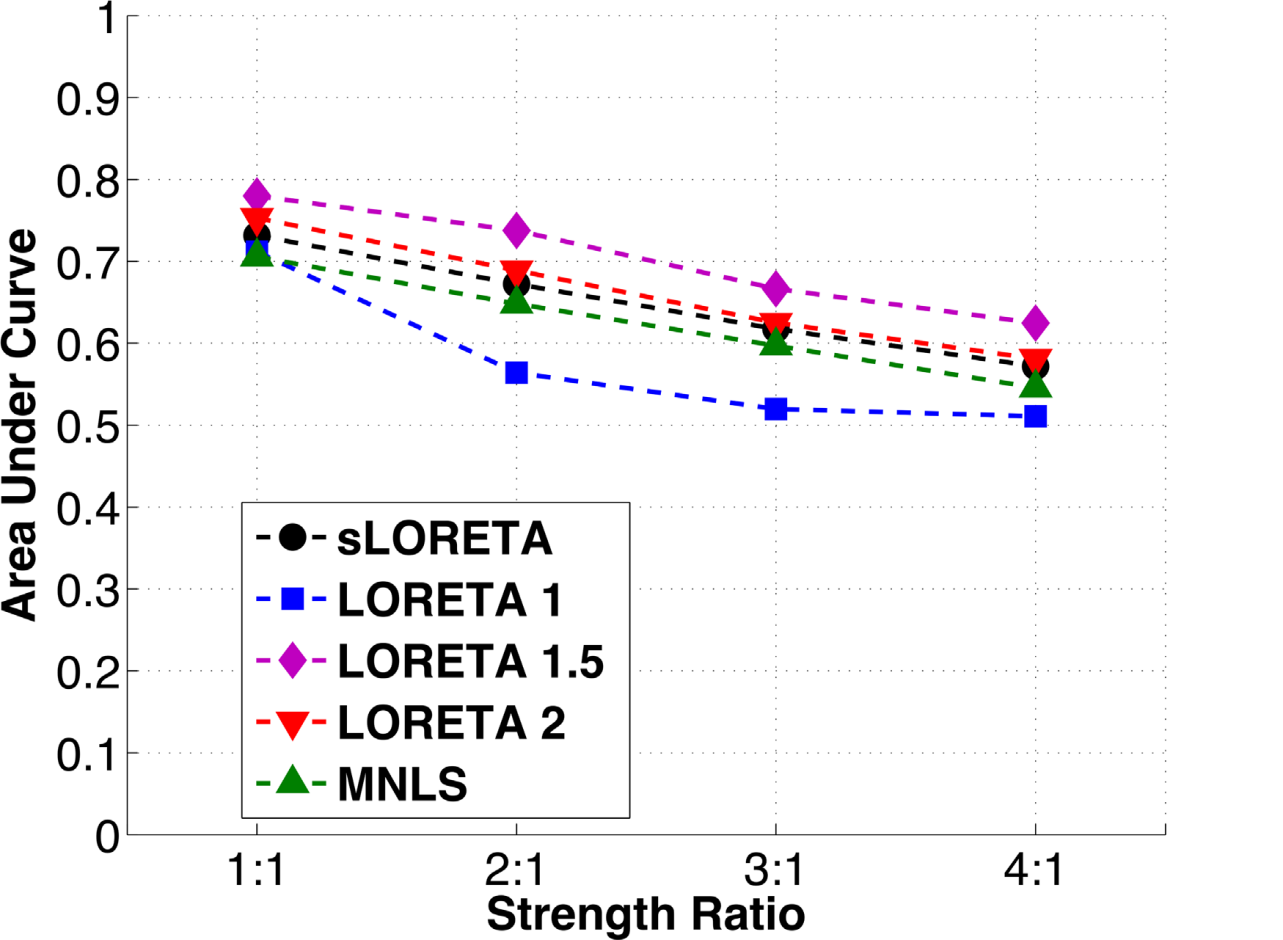
Area under the Precision vs. Recall curve for each of the algorithms tested vs. strength ratio. As a summary of the performance of the algorithm, the area under the curve decrease slightly for all algorithms as the strength ratio of the sources increases.

The relative strengths of the two simulated sources were varied to determine if any of the algorithms tested performed better or worse than the other algorithms under this condition. Again, all the algorithms had similar Precision-Recall curves. As the strength ratio increased from 1:1 to 4:1, peak Precision results changed only slightly, while Recall drops off sharply (Fig 5A–5D). This is reflected in decreases in the area under the Precision-Recall curve with increasing strength ratio (Fig 6). LORETA 1.5 continues to have higher AUC than other algorithms when the strength ratio between the two sources is increased, and this difference is statistically significant (Table 1).

## Discussion

The results of the simulation experiments presented in this paper provide guidance regarding the performance of a number of source localization algorithms in distinguishing 1–4 cortical sources. It is the first study that quantifies the performance of these source localization algorithms in the presence of more than two active cortical areas and sources of different strengths.

While ideally a CSD algorithm would locate all sources (i.e. have 100% Recall) without falsely identifying cortical areas as active (i.e. have 100% Precision), in reality that is rarely the case when more than two areas are active. For example, for a 90% chance of finding two sources (Recall of 0.9), the highest Precision value possible (for LORETA 1) is 0.4, meaning only 40% of the represented activity corresponds to “real” activity. These figures should serve as a caution for those interpreting the results of these algorithms: If multiple distinct areas are identified as active in CSD results, it is unlikely that all of these areas correspond to “real” activity. Conversely, when only a small area is identified as active it is possible that other sources of activity are being missed.

Note that these simulation results are based on signals with relatively high signal to noise ratio (SNR = 10), and the source space and head model are identical. In practice, higher noise and inaccuracies in the head model are likely to further reduce the Precision and Recall [22, 23]. A smaller number of electrodes would also likely reduce the accuracy of results, although previous studies suggest a plateau in performance above 100 electrodes [1]. In addition, we chose a distance threshold of 10 mm as our margin for counting a source as correct, which may be too large for some CSD purposes. This study also did not assess if closely spaced sources were resolved. Although not a focus of this study, the issue of CSD resolution has previously been addressed by Yao and Dewald (2005) [15].

On the other hand, source locations were chosen at random locations on the cortex that may not correspond to real source locations, and Precision and Recall varied depending on the position of the source on the cortex. It is therefore possible that the Precision and Recall for a particular source configuration may be higher than those reported here. Simulations of expected source distributions could be used to determine the accuracy expected for those particular source locations. Additionally, no constraint of sources to a region of interest or weighting towards the likely locations of the sources was used in the reconstructions. If accurate, a priori information is likely to improve the performance of source localization results (e.g. [24–28]).

Note that it is possible to find one source with high Precision (>0.9 for sLORETA), even when three other sources are also active. When these sources are of uneven strengths, the strongest source is the one most likely to be found. For many experiments, this may be sufficient–if a hypothesis only involves the most prominent of sources, locating that source accurately may be enough. sLORETA has by far the best performance for one source, as previously reported [14]. The sLORETA algorithm includes information on the uncertainty in the data in its calculation of the source distribution that the other algorithms do not, which appears to be beneficial. While non-CSD methods, such as dipole analysis may be appropriate when only one source is expected, the accuracy of dipole methods has previously been found to be lower than for CSD methods [15].

If reconstructing more than one source is a priority, sLORETA is less likely to find all sources than other algorithms. LORETA 1.5 maintains Precision over a wider range of Recall, and is therefore more likely to reconstruct all sources with higher Precision.

We chose to use white noise in our simulations rather than more realistic, physiological noise as the effect of source strength on source localization was also being tested and physiological noise may contain additional low strength active sources. Comparing the results with one source (Fig 3A) to the results with two sources with a strength ratio of 4:1 (Fig 5D), it can be seen that the addition of the low strength source minimally affects the peak precision of finding the first source, and the order of algorithm performance is the same. We therefore would not expect results with the addition of more realistic noise to be substantially different than the results presented in this article. Additionally, the fact that precision results are lower for two sources with a 4:1 ratio than that for one source alone indicates that the second, low strength source is not represented in the CSD results. Therefore, if a second source is four times smaller than the most prominent source, it should not be expected to be localized.

Peak Precision seems to be higher for two or more sources than for one source for many of the algorithms. This can be explained if we consider that every reconstruction will have a certain amount of randomly assigned activity (noise). If that noise falls within 10 mm of the “real” source, it will be counted as correct. When there are more sources, more area of the cortex counts as correct, therefore the overall Precision increases.

The poor performance of the LORETA 1 algorithm was somewhat unexpected, as that algorithm had outperformed all other algorithms tested in a previous study [15]. In that study, LORETA 1 had lowest location error distance for one and two simulated sources. LORETA 1 reconstructions generally have more focused activity than other algorithms, which often have some areas of focus and other areas of sparse activity. This focused activity is beneficial when calculating error distances, as all the activity is focused in one area, usually close to the simulated source. This focused activity is also beneficial when resolving two closely spaced sources, as was the case in the paper by Yao and Dewald (2005) [15]. However, for widely separated sources, as in this study, the tendency to focus activity in one area is more problematic, and leads to a reduced probability of finding all of the sources.

There are some limitations to these results. Although realistic sources may be larger than the 5mm radius areas simulated here, a previous study found no relationship between the size of the source and the accuracy of the reconstruction [6], and our own preliminary unpublished results with larger sources (8 mm radius / 25 dipoles) showed little difference in the Precision and Recall found (**S1 Fig).** We therefore continued with 5 dipole simulations to simplify the simulation process. The effect of other parameter choices, such as the regularization parameter and the method of choosing it are unknown, but may influence results.

Finally, a limited number of source localization algorithms were tested in this study. LORETA, MNLS and sLORETA are among the most common currently used source localization algorithms, and are widely available and accessible. LORETA with p-norms of 1 and 1.5 are less common, but were included in this analysis as they had performed well on previous tests [15]. While other, more recently developed, source localization algorithms may outperform those tested here for the localization of multiple sources (e.g. [29–34]), those algorithms were not available to us in an implemented form. In particular, incorporating information on the temporal dynamics of the EEG signal into the source localization process is a promising area of research, as in real recording situations sources tend to be active over more than one time point (e.g. [29–31, 34]). We hope that the data presented here can be used as a benchmark for the developers of source localization algorithms, as all code and simulated data are available at the online depository corresponding to this article [35].

## Conclusion

If accurately locating only the strongest source is sufficient, sLORETA is an appropriate choice of source localization algorithm. However, if accurately locating more than one source is a priority, LORETA with a p-norm with p equal to 1.5 is recommended. If possible, using a combination of algorithms may be useful. For example, sLORETA could be used to accurately locate the most prominent source, and LORETA 1.5 could then be used to find other sources. In any case, we advise researchers to be aware of the probability that their reconstruction is accurate and complete when interpreting results of source localization algorithms.

## Supporting Information

S1 Fig Source size comparison. Precision vs, Recall for each source localization algorithm tested for (a) one small (1 dipole), (b) one medium sized (5 dipole) and (c) one large (30 dipole) simulated source.
(PDF)
