# Supplementary material for: Evaluation of Electroencephalography Source Localization Algorithms with Multiple Cortical Sources
Source: PLoS One. 2016 Jan 25;11(1):e0147266. doi: 10.1371/journal.pone.0147266 (PMC4725774; doi:10.1371/journal.pone.0147266)
Supplement: S1 Fig — Precision vs, Recall for each source localization algorithm tested for (a) one small (1 dipole), (b) one medium sized (5 dipole) and (c) one large (30 dipole) simulated source. (PDF) [file pone.0147266.s001.pdf]

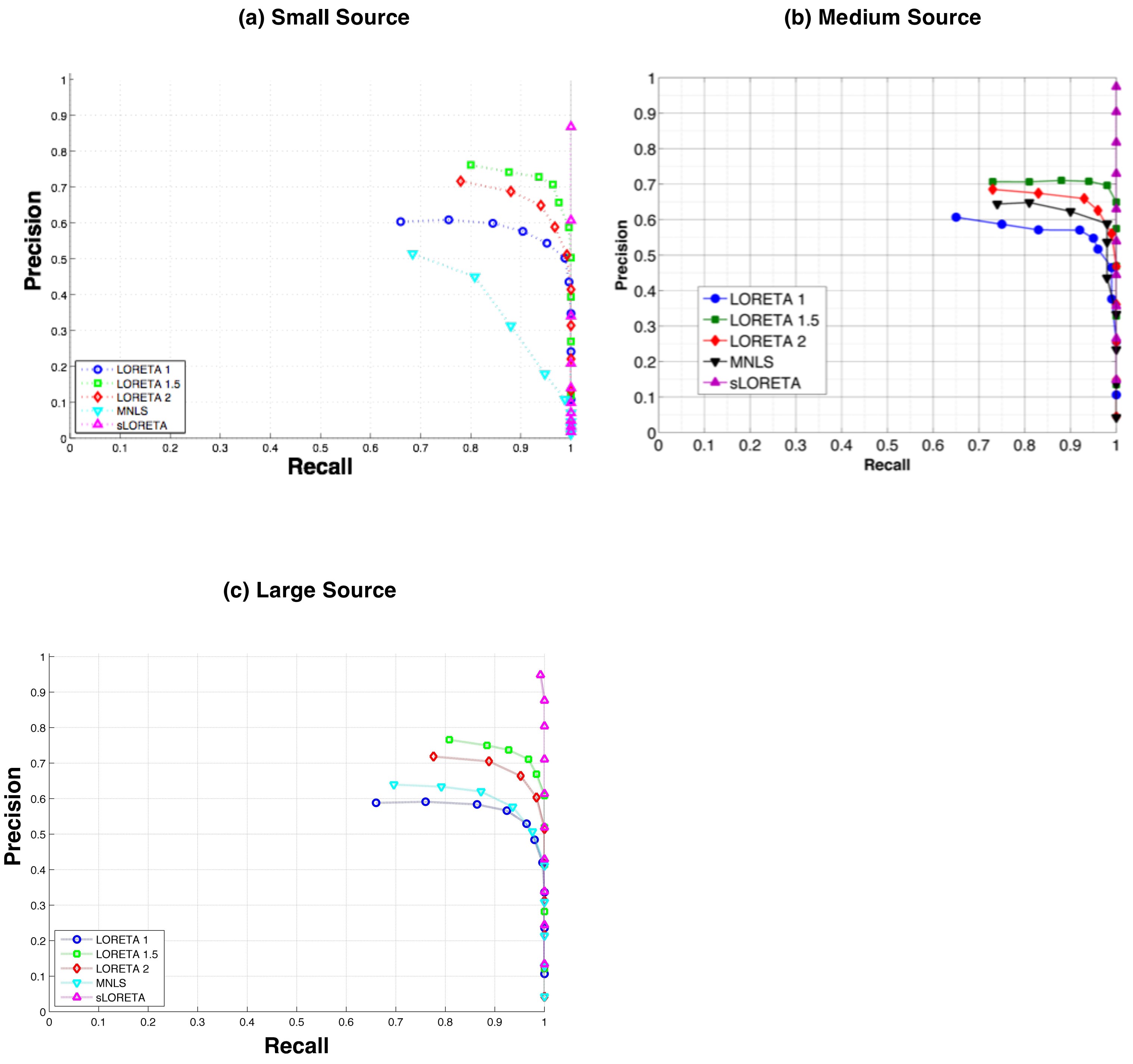

***Figure S1: Source size comparison**. Precision vs, Recall for each source localization algorithm tested for (a) one small (1 dipole) (b) one medium sized and (b) one large (30 dipole) simulated source.*
